# Recent advances in understanding eosinophil biology

**DOI:** 10.12688/f1000research.11133.1

**Published:** 2017-07-07

**Authors:** Amy Klion

**Affiliations:** 1 Human Eosinophil Section, Laboratory of Parasitic Diseases, National Institutes of Allergy and Infectious Diseases, National Institutes of Health, Bethesda, MD, USA

**Keywords:** eosinophils, life cycle, heterogeneity, cell-cell communication

## Abstract

With the advent of novel therapies targeting eosinophils, there has been renewed interest in understanding the basic biology of this unique cell. In this context, murine models and human studies have continued to highlight the role of the eosinophil in homeostatic functions and immunoregulation. This review will focus on recent advances in our understanding of eosinophil biology that are likely to have important consequences on the development and consequences of eosinophil-targeted therapies. Given the breadth of the topic, the discussion will be limited to three areas of interest: the eosinophil life cycle, eosinophil heterogeneity, and mechanisms of cell-cell communication.

## Introduction

Eosinophils are primitive myeloid cells found in all vertebrate species, including zebrafish ^1^. Historically, eosinophils have been viewed as effector cells involved primarily in the defense against parasites and in allergic inflammation, but the role of these cells in homeostasis and immunoregulation has become increasingly clear over the past decade ^2, 3^. This is due in large part to the development of several strains of eosinophil-deficient mice ^4– 6^, which have been instrumental in demonstrating a role for murine eosinophils in a wide variety of fundamental processes, including antibody production ^7, 8^, glucose homeostasis ^9^, and muscle and liver regeneration ^10, 11^. Although preliminary findings corroborate a role for eosinophils in many of these processes in humans ^7, 8, 12, 13^, definitive data have been elusive. In this regard, the recent explosion in the development of novel therapies that deplete eosinophils or affect eosinophil function ^14, 15^ provides a unique opportunity to increase our understanding of the role of this multifunctional and complex cell in human health and disease.

A comprehensive list of all of the important recent findings in eosinophil biology is beyond the scope of this review. Consequently, this article will focus on three aspects of eosinophil biology in which there have been major advances in the past several years: (1) the eosinophil life cycle, (2) eosinophil heterogeneity, and (3) mechanisms of cell-cell communication. Each of these has important implications for eosinophil-targeted therapies, especially since the long-term consequences of eosinophil depletion will depend not only on the efficacy of depletion of eosinophils but also on the processes that are perturbed in their absence.

## The eosinophil life cycle

### Eosinophilopoiesis

As is true of other circulating leukocytes, eosinophils differentiate from CD34 ^+^ progenitor cells in the bone marrow under the influence of a variety of lineage-specific and common transcription factors and cytokines (reviewed in 16). Beginning in the late 1980s with the discovery of the critical roles of the cytokine, interleukin-5 (IL-5), and the GATA transcription factors ^17– 19^, the delineation of the sequential steps involved in eosinophilopoiesis has been instrumental in the development of a number of innovative mouse models to study eosinophilic disorders. These include the ΔdblGATA-1 and PHIL eosinophil-less mice ^4, 5^ and the recently described MBP-1/EPX double-knockout eosinophil-less mouse ^6^ and Cre recombinase eosinophil transgenic mouse ^20^. A new addition to this cohort is the recently described *Xbp1*-null mouse, in which deletion of the transcription factor, *Xbp1*, in multi-lineage hematopoietic progenitor cells causes a lineage-restricted late maturational arrest in eosinophil development (due at least in part to dysregulated production and assembly of granule proteins) and a total absence of circulating eosinophils ^21^. Since early eosinophilopoiesis is unaffected in the *Xbp1*-null mouse and eosinophil precursors (EoPs) appear to be normal, this model is likely to provide a unique window into the role of eosinophil granule protein packaging in the terminal differentiation of eosinophils. *Xbp1* may also prove to be a novel lineage-specific therapeutic target for the treatment of eosinophilic disorders.

New transcription factors involved in the negative regulation of eosinophil development have also been recently identified. These include RhoH—a negative regulator of eosinophilopoiesis that is upregulated by IL-5, IL-3, and granulocyte-macrophage colony-stimulating factor (GMCSF) and likely functions through GATA2 ^22^—and Olig2, which is expressed late in eosinophil development and regulates expression of Siglec-8, an inhibitory receptor restricted to eosinophils, basophils, and mast cells ^23^. Finally, a more global approach using genome-wide transcriptome and epigenome analysis has both confirmed prior findings and led to the identification of previously unreported transcriptional regulators of eosinophil development, including Helios and Aiolos ^24^.

EoPs were first identified in the peripheral blood and nasal mucosa of atopic subjects in the late 1980s by using colony-forming assays ^25, 26^. These results were confirmed by many different groups using flow cytometry, immunohistochemical staining, or *in situ* hybridization (or a combination of these) to identify CD34 ^+^, and more recently CD34 ^+^IL-5Rα ^+^, cells in the blood and tissues of patients with allergic disorders ^27– 30^. Increased circulating levels of EoPs have also been described in patients with active eosinophilic esophagitis (EoE), a food antigen–driven eosinophilic disorder ^31^. The clinical relevance of these findings is supported by the correlation of EoPs in blood and sputum with disease activity ^27, 31, 32^.

Whereas the acquisition of IL-5Rα on the surface of CD34 ^+^ cells has long been recognized as a critical event in the expansion and maturation of EoPs ^33, 34^, the factors driving eosinophil lineage commitment are less well understood. Recent data suggest that IL-33 may play a significant role in this process. IL-33 and its receptor, ST2, were first described in 2005 ^35^. Although a role for IL-33 in the induction of IL-5 and eosinophilia was first proposed at that time, these effects were attributed to the production of IL-5 by Th2 cells. Eosinophil expression of ST2 was subsequently demonstrated, suggesting that IL-33 might also interact directly with eosinophils ^36^. In their recent report, Johnston *et al*. have taken this one step further, demonstrating in a mouse model that ST2 is expressed on EoPs and that IL-33 both expands the EoP compartment and upregulates IL-5Rα expression on EoPs ^37^. The authors conclude that IL-33 precedes IL-5 in regulating lineage commitment in eosinophils and that this is important in maintaining eosinophil homeostasis. In a separate study, Anderson *et al*. showed that IL-5 and IL-33 produced in the lung (but not bone marrow) in a murine model of Alternaria exposure lead to increased numbers of eosinophils and EoPs in bone marrow ^38^, suggesting that IL-33 also plays an important role in allergen-induced eosinophilia. The capacity of IL-33 to directly induce murine EoP production of Th2 and inflammatory cytokines associated with allergic inflammation has also been reported ^39^. These data provide a potential explanation for the observation that anti–IL-5 treatment with mepolizumab depletes blood eosinophils but not their precursors in the bone marrow of patients with asthma ^40^. Of note, mepolizumab treatment did lead to a decrease in CD34 ^+^IL-5Rα ^+^ cells in bronchial mucosal biopsies from the same patients, suggesting potential differences in IL-5 dependence between lung and bone marrow EoPs.

Whereas the IL-33/ST2 axis clearly plays an important role in the regulation of bone marrow eosinophilia, recent data describing the opposing roles of paired immunoglobulin-like receptor A (PIR-A) and PIR-B on IL-5–induced eosinophil development illustrate the complexities of this process and the implications for eosinophil-associated pathogenesis ^41^. Using a murine model, the authors demonstrated that PIR-B, in the context of self-recognition through major histocompatibility complex (MHC) class I, allowed IL-5–induced eosinophilopoiesis by blocking the pro-apoptotic activity of PIR-A. Importantly, mice lacking PIR-B had decreased lung eosinophilia in response to aeroallergen challenge. Eosinophil expression of the human homologues of PIR-A and PIR-B, leukocyte immunoglobulin-like receptors B1 and B2 (LILRB1 and LILRB2), has been described ^42^, although their function has not been studied to date.

Many of the transcription factors and cytokines involved in eosinophilopoiesis have also been shown to play a role in the development of other lineages, most notably basophils and mast cells. This has important implications for eosinophil-targeted therapies, which may or may not deplete multiple cell types. In an elegant study using reporter mice expressing enhanced fluorescent green protein from GATA-1 and single-cell sequencing, Drissen *et al*. demonstrated that eosinophils, mast cells, and likely basophils (although this was not examined directly) are generated from a dedicated progenitor that arises prior to the segregation of the erythroid-megakaryocytic and lymphoid lineages, rather than from a common myeloid precursor ^43^. The applicability of these data to the human system awaits confirmation.

### Eosinophil trafficking

Since tissue eosinophilia is integral to the pathogenesis of a wide variety of eosinophilic disorders, the mediators involved in eosinophil trafficking to the tissue during inflammation provide ideal therapeutic targets. Most early interest focused on eotaxins (CCL11, CCL24, and CCL26) and their receptor CCR3 because of their role in promoting tissue eosinophilia in a wide variety of eosinophilic disorders, including asthma, eosinophilic gastrointestinal (GI) disease, eosinophilic skin diseases, and most recently eosinophil trafficking to the heart in a murine model of myocarditis ^44^. Despite promising pre-clinical data, however, a clinical trial with an oral CCR3 antagonist was ineffective in reducing sputum eosinophilia in patients with asthma ^45^.

IL-13 is known to promote eotaxin production by a variety of cell types. Consequently, therapies targeting IL-13 would be expected to block migration of eosinophils from the bloodstream to the tissue. Consistent with this hypothesis, clinical trials of monoclonal antibodies to IL-13 or its receptor in patients with asthma have consistently shown increases in peripheral blood eosinophil counts ^46– 48^, and tissue eosinophilia was decreased in a recent phase 2 study of anti–IL-13 antibody in EoE ^49^. Unfortunately, the effects on eosinophil numbers were mild to moderate in most of these studies, suggesting that the IL-13/eotaxin/eosinophil connection may be more complex than previously thought.

The recent demonstration that the inhibitory receptor, PIR-B, is involved in preventing IL-13–induced esophageal eosinophilia in a murine model of EoE provides an important example in support of this conclusion ^50^.

### Eosinophil senescence

Although the role of cytokines and other mediators in eosinophil survival has been recognized for more than 30 years, very little is known about the mechanisms by which these pro-survival cues determine the eosinophil life span. The recent description of a long non-coding RNA, *Morrbid*, which enables allele-specific control of pro-apoptotic gene transcription in response to extracellular cytokine signals, begins to provide an answer to this very basic question ^51^. Initially described in mice, *Morrbid* expression was increased in eosinophils from patients with hypereosinophilic syndromes and correlated with serum IL-5 levels, suggesting that *Morrbid* plays a similar regulatory role in human eosinophils and may be a novel target for therapeutic intervention. As mentioned in the Eosinophilopoiesis section, our understanding of the regulation and function of inhibitory receptors, including Siglec-8 ^23, 36, 52, 53^, which are important in eosinophil apoptosis, has also advanced significantly in recent years. This has led to the development of novel therapeutic agents for eosinophilic disorders, including two monoclonal antibodies to Siglec-8 that are currently in clinical trials for nasal polyposis and systemic mastocytosis in Europe.

## Eosinophil heterogeneity

Eosinophil heterogeneity was first proposed in the early 1980s with the description of hypodense eosinophils in the blood of patients with eosinophilia of varied etiologies, including allergic disease, helminth infection, and idiopathic hypereosinophilic syndrome ^54– 56^. The reproduction of this phenomenon *in vitro* and its association with enhanced eosinophil cytotoxic activity and degranulation were described shortly thereafter ^57, 58^. Since that time, numerous studies have confirmed the ability of a wide variety of activating stimuli to induce degranulation and a “hypodense” phenotype. Whether all hypodense eosinophils are functionally equivalent despite differences in the activating stimulus remains unclear.

The first evidence that tissue eosinophils might be different from blood eosinophils came from studies in the 1980s comparing density and respiratory burst in blood and bronchoalveolar lavage eosinophils from individuals with pulmonary eosinophilia ^59^. Subsequent studies have demonstrated changes in expression of surface receptors, including IL-5Rα and integrins, on eosinophils recruited to the lung following segmental allergen challenge ^60– 62^. More recently, several groups have demonstrated associations between specific surface phenotypes and eosinophil function in murine models of allergic inflammation in the lung. In one such study, Mesnil *et al*. characterized two distinct eosinophil subsets in the lungs of mice following allergen challenge ^63^. These subsets, resident (rEos) and inflammatory (iEos) eosinophil, differed not only in location (parenchymal versus peribronchial), nuclear morphology (ring-shaped versus segmented), surface phenotype (Siglec-F ^int^CD62L ^+^CD101 ^lo^ versus Siglec-F ^hi^CD62L ^−^CD101 ^hi^), and dependence on IL-5 (independent versus dependent) but also in their ability to down-modulate the inflammatory response ^63^. Although similar populations have not been functionally characterized in humans, parenchymal rEos in non-asthmatic lungs displayed a different surface phenotype than iEos in the sputa of patients with eosinophilic asthma ^63^. Whether the presence of IL-5–independent rEos might explain the clinical efficacy of anti–IL-5 antibody therapy in asthma, despite the persistence of tissue eosinophilia ^64^, remains to be seen. Using a different set of surface markers (Siglec F and Gr1), Percopo *et al*. identified two morphologically similar eosinophil populations with different cytokine profiles in mouse lung following allergen challenge ^65^.

The GI tract provides another example of the potential differences between tissue and blood eosinophils. Eosinophils are normal residents of the lamina propria of the GI tract and, unlike the situation in other tissues, appear to undergo degranulation under homeostatic conditions ^66, 67^. The relevance of this finding has become increasingly clear with the demonstration that eosinophils in the GI tract play important roles in mucosal immunity, including the promotion of IgA class switching and the maintenance of IgA plasma cells ^8, 68, 69^. Although eosinophils increase in the intestine in a number of pathological settings—including intestinal nematode infection, inflammatory bowel disease, and eosinophilic GI disorders—and clearly can contribute to tissue inflammation and damage, recent data from several groups suggest that they may help limit tissue destruction and preserve the intestinal barrier in some settings. Examples include the requirement for eosinophils in the suppression of Th2 responses in Peyer’s patches during an intestinal nematode infection in mice ^70^ and for IL-25–mediated maintenance of the intestinal barrier during murine infection with *Clostridium difficile* infection ^71^. Of note, similarities between IL-25 expression in intestinal biopsies in mice and humans with *C. difficile* infection suggest that eosinophils may also be important in the maintenance of tissue integrity in human infection ^72^. These protective functions of eosinophils in GI disease seem to be more pronounced in the tissue. In support of this, recent studies have demonstrated that murine lamina propria eosinophils, but not blood eosinophils, are able to induce differentiation of naïve T cells into regulatory T cells *in vitro* ^72^ and suppress differentiation of Th17 cells through production of IL-1Ra ^73^. Although abnormal responses to infection have not been reported in patients receiving eosinophil-depleting therapies, vigilance is needed as these therapies reach a wider population with exposure to helminths and other infectious agents.

## Cell-cell communication

Eosinophils have a complex subcellular structure that includes primary and secondary granules, lipid bodies, and a dynamic intracellular vesicular system ^74^. Moreover, contained within their secondary granules are cationic granule proteins and a host of preformed cytokines and other soluble mediators that can be rapidly mobilized for secretion in response to a wide variety of stimuli ^75^. Although a variety of secretory processes have been described (piecemeal degranulation, exocytosis, and cytolysis), the mechanisms by which eosinophils selectively secrete mediators are incompletely understood and remain an active area of research. Some of the more exciting recent advances in this field are described below.

### Surface receptors

CD63 is a member of the transmembrane-4 glycoprotein superfamily (tetraspanins) that is found on the surface of secretory granules in multiple cell types, including eosinophils. Translocation of CD63 to the eosinophil cell surface during piecemeal degranulation was first demonstrated in 2002 ^76^. Recent studies using CD63 labeling provide evidence for distinct CD63-dependent secretory processes in eosinophils depending on the stimulus provided (piecemeal degranulation in response to CCL11 versus compound exocytosis in response to tumor necrosis factor alpha, or TNFα) ^77^.

### Eosinophil-derived extracellular DNA traps

Eosinophil-derived extracellular DNA traps (EETs) containing eosinophil granules were first described in 2008 in the GI tract, where they were believed to play a primary role in antibacterial defense ^78^. Although these initial EETs contained mitochondrial DNA that was catapulted from an intact cell, subsequent studies have demonstrated that eosinophils can also release nuclear DNA into the extracellular space during cytolysis (ETosis) ^79^. EETs have been described in a variety of tissues, including skin, lung, and the GI tract, where their presence has been correlated with disease activity ^80^. More recently, EETs have been identified in eosinophil-rich secretions from patients with eosinophilic rhinosinusitis and otitis media ^81^. Ultrastructural analysis of ultrathin sections using transmission electron microscopy demonstrated globular chromatin fibers containing intact eosinophil granules, providing a potential explanation for persistent effects of eosinophilic inflammation once the eosinophil itself is no longer present.

### Exosomes

Secreted microvesicles that are believed to function in extracellular communication, exosomes have recently been demonstrated in the culture supernatants of human eosinophils ^82, 83^. As expected, eosinophil exosomes express CD63 and CD9 on their surface and contain a variety of eosinophil proteins, including eosinophil cationic protein and eosinophil peroxidase ^84^. Eosinophil production of exosomes was increased in patients with asthma and in response to *in vitro* stimulation with CCL11, TNFα, or interferon gamma ^82, 83^. Moreover, eosinophil-derived exosomes induced an increase in reactive oxygen intermediates by eosinophils and could induce eosinophil adhesion and chemotaxis *in vitro* ^84^. Since the composition of eosinophil exosomes appears to be similar between asthmatic patients and normal controls, these data suggest that selective packaging of mediators into exosomes may play an important role in modulating the outcomes of eosinophil activation in different settings.

## Conclusions and therapeutic implications

The past several years have seen the publication of the eosinophil transcriptome, proteome, and epigenome ^85– 88^. These tools, coupled with the availability of innovative mouse models and increasing numbers of targeted therapies in humans, are likely to dramatically increase our understanding of the basic biology of eosinophils and their role in a wide variety of disorders associated with blood and tissue eosinophilia. Conversely, unraveling the complexity of the eosinophil and its role in homeostasis and pathogenesis will certainly lead to the identification of novel therapeutic agents, as evidenced by the development of monoclonal antibodies to IL-5 and IL-5 receptor for the treatment of eosinophilic asthma and other eosinophilic disorders. Despite these advances, a number of questions remain, including the morphological and functional features that define eosinophil activation, the relationship of these features to disease pathogenesis, and the long-term safety of therapies that target eosinophils, particularly those that deplete eosinophils more completely in the tissues than currently approved agents and those that concomitantly target additional lineages, including mast cells and basophils. The interactions between eosinophils and other cells in the bone marrow, blood, and tissues are key factors in this regard. Negotiating the balance between eosinophil-driven pathogenesis and maintenance of homeostasis will be the next major challenge.

## Abbreviations

EET, eosinophil-derived extracellular DNA trap; EoE, eosinophilic esophagitis; EoP, eosinophil precursor; GI, gastrointestinal; iEos, inflammatory eosinophil; IL, interleukin; PIR, paired immunoglobulin-like receptor; rEos, resident eosinophil; TNFα, tumor necrosis factor alpha.
